# Age is not the only risk factor in COVID-19: the role of comorbidities and of long staying in residential care homes

**DOI:** 10.1186/s12877-021-02013-3

**Published:** 2021-01-15

**Authors:** M. D’ascanio, M. Innammorato, L. Pasquariello, D. Pizzirusso, G. Guerrieri, S. Castelli, A. Pezzuto, C. De vitis, P. Anibaldi, A. Marcolongo, R. Mancini, A. Ricci, S. Sciacchitano

**Affiliations:** 1 Division of Pneumology, Sant’Andrea Hospital, Via di Grottarossa, 1035-1039, 00189 Rome, Italy; 2 Department of Clinical and Molecular Medicine, Sapienza University, Viale Regina Elena n. 324, 00161 Rome, Italy; 3 Health Director, Sant’Andrea Hospital, Via di Grottarossa, 1035-1039, 00189 Rome, Italy; 4 General Director, Sant’Andrea Hospital, Via di Grottarossa, 1035-1039, 00189 Rome, Italy; 5 Laboratory of Biomedical research, Niccolò Cusano University Foundation, Via Don Carlo Gnocchi, 3, 00166 Rome, Italy

**Keywords:** Elderly, Comorbidities, COVID 19, Residential care homes

## Abstract

**Background:**

The actual SARS-CoV-2 outbreak caused a highly transmissible disease with a tremendous impact on elderly people. So far, few studies focused on very elderly patients (over 80 years old). In this study we examined the clinical presentation and the outcome of the disease in this group of patients, admitted to our Hospital in Rome.

**Methods:**

This is a single-center, retrospective study performed in the Sant’Andrea University Hospital of Rome. We included patients older than 65 years of age with a diagnosis of COVID-19, from March 2020 to May 2020, divided in two groups according to their age (Elderly: 65–80 years old; Very Elderly > 80 years old). Data extracted from the each patient record included age, sex, comorbidities, symptoms at onset, the Pneumonia Severity Index (PSI), the ratio of the partial pressure of oxygen in arterial blood (PaO2) to the inspired oxygen fraction (FiO2) (P/F) on admission, laboratory tests, radiological findings on computer tomography (CT), length of hospital stay (LOS), mortality rate and the viral shedding.

The differences between the two groups were analyzed by the Fisher’s exact test or the Wilcoxon signed-rank test for categorical variables and the Mann-Whitney U test for continuous variables. To assess significance among multiple groups of factors, we used the Bonferroni correction.

The survival time was estimated by Kaplan-Meier method and Log Rank Test. Univariate and Multivariate logistic regression were performed to estimate associations between age, comorbidities, provenance from long-stay residential care homes (LSRCH) s and clinical outcomes.

**Results:**

We found that Very Elderly patients had an increased mortality rate, also due to the frequent occurrence of multiple comorbidities. Moreover, we found that patients coming from LSRCHs appeared to be highly susceptible and vulnerable to develop severe manifestations of the disease.

**Conclusion:**

We demonstrate that there were considerable differences between Elderly and Very Elderly patients in terms of inflammatory activity, severity of disease, adverse clinical outcomes. To establish a correct risk stratification, comorbidities and information about provenience from LSRCHs should be considered.

## Background

The COVID 19 outbreak represents an historically unprecedented pandemic, particularly dangerous and potentially lethal for elderly population. Since the beginning of the actual SARS-CoV-2 outbreak it was evident that older people, compared to younger ones, were at higher risk to get the infection and to develop a more severe disease with unfavorable prognosis. In Italy, the National Healthcare Service, registered 418,142 total cases of COVID-19 and 39,412 deaths, as of November 3, 2020 [1]. The mean age of patients that died was 80 years, at least 20 years higher than that registered in infected people. The majority of those who are infected, that have a self-limiting infection and do recover are, in fact, younger. Conversely, those that suffer a more severe disease, that require intensive care unit admission and eventually pass away are older [2, 3].

Many reports indicate that elderly patients with COVID-19 are more likely to progress to severe disease compared to young and middle-aged ones [2]. These observations have a particular and relevant negative impact in Europe. Of the top 30 countries with the largest percentage of older people, all but one (Japan) are States in Europe and Italy is considered “a country of old people” because the life expectancy is over 80 years of age [4]. The situation is critical in the long-stay residential care homes (LSRCHs), where many elderly people and people with disabilities and severe cardiovascular and neurological diseases live all together in close contact, facilitating the dynamics of virus transmission. Residents in LSRCHs are a vulnerable population group and it has been reported that the proportion of COVID-19 cases who have died in these LSRCHs has exceeded 60% of all reported deaths [5].

In a recent report by the Organization for Economic Cooperation and Development (OECD) [6] over 75,000 deaths have been registered across 13 European countries amongst residents in long term care institutions (as of early October 2020), accounting for almost half of the total deaths caused by COVID-19. In Italy there are more than 3000 of such facilities, with 186,872 beds. That is why the Istituto Superiore di Sanità (ISS) launched a National survey on COVID-19 infection in LSRCHs, to monitor the situation and to solicit special strategies against spreading of infection [7]. The results of this Survey have been described in a final report posted on May 5th 2020 [8]. The data obtained from a total of 3292 LSRCHs in different Italian regions indicated that the mortality rate of patients, with proved infection plus those with symptoms that suggested positivity, was equal to 3.1%, but, in some regions, it reached the value of 6.5%. The main problems reported by the personnel in facing the pandemic were related to the difficulties in isolating the positive patients and in transfer the critical ones to the hospital. In addition, they complained about the scarcity of tests, adequate information and personal protective equipment.

However, despite the huge amount of data on COVID-19 worldwide, the reasons why older people are at significantly increased risk of severe disease following infection from COVID-19 are not clear. Age could not be the only risk for severe disease. It has been reported that people in their teens or twenties may develop a severe form of the disease, may require intensive care and may die as well [9]. On the other side, people who age healthily seems to be less at risk. Besides the physiological changes associated with ageing, other factors could be involved, including the decreased immune function and the occurrence of multimorbidity [10].

To date there is little information on COVID-19 in patients over 80 years of age. Therefore, in this study, we focused our attention on very elderly population (> 80 years of age) [11, 12] that referred to our University Hospital during the actual SARS-CoV-2 outbreak and we have analyzed the differences between elderly people and very elderly ones. Our aim was to try to figure out the reason why elderly and very elderly people are more prone to be infected and to develop a more severe form of the disease. We considered clinical aspects, presence of comorbidities, laboratory and radiological findings as well as their outcome, including the length of stay, mortality and viral shedding in nasal/oropharyngeal swabs.

## Methods

### Study design and participants

This is a single-center, retrospective study performed in the Sant’Andrea University Hospital of Rome. We included patients older than 65 years of age with a diagnosis of COVID-19 from March 2020 to May 2020. The diagnosis was based on the Chinese Clinical Guidance For COVID-19 Pneumonia Diagnosis and Treatment published and updated by the National Health Commission of China (NHFPC, 2020). All the COVID-19 patients had either positive real-time reverse transcription polymerase chain reaction (rRT-PCR) from respiratory samples [13] or positive serological test of specific IgM antibody to SARS-CoV-2. Eighty-one patients were divided into two groups according to their age. Forty-one patients with ages ranging from 65 to 79 years were included in Elderly Group, the remaining forty patients with ages > 80 years were included in group named Very Elderly.

Data extracted from the each patient record included age, sex, comorbidities, symptoms at onset, the Pneumonia Severity Index (PSI), the ratio of the partial pressure of oxygen in arterial blood (PaO2) to the inspired oxygen fraction (FiO2) (P/F) on admission, laboratory tests, radiological findings on computer tomography (CT), length of hospital stay (LOS), mortality rate and the viral shedding.

### Serum markers of inflammation and sepsis

We analyzed several different serum markers of systemic inflammation and sepsis, including the neutrophil to lymphocyte ratio (NLR), the Platelets to Lymphocytes Ratio (PLR), the pro-calcitonin (PCT) and the high-sensitive C-Reactive Protein (hs-CRP).

### Radiological evaluation and analysis

Chest CT were obtained on a 128-slice scanner (GE Revolution EVO CT Scanner, GE Medical Systems, Milwaukee, WI, USA), with patients in supine position and during end-inspiration, without iodinated contrast medium injection. The following technical parameters were used: tube voltage: 120 kV; tube current modulation: 100–250 mAs; spiral pitch factor: 0.98; collimation width: 0.625. Images were reconstructed with a sharp convolution kernel (BONEPLUS) at a slice thickness of 1.25 mm.

DICOM data have been transferred onto a PACS workstation (Centricity Universal Viewer v.6.0, GE Medical Systems, Milwaukee, WI, USA) and independently evaluated by two expert radiologists, using a dedicated software (Thoracic VCAR v13.1, GE). Only imaging features related to COVID-19, according to the most recent literature [14, 15] have been considered valid for image analysis.

### Outcomes

Length of stay and death were both analyzed.

During hospitalization, an RT-PCR assay was conducted every other day after the remission of clinical symptoms or radiography of patients, and the corresponding date was recorded. The duration of viral shedding was defined as the number of days from the onset of the symptoms until the successive negative detection of SARS-CoV-2 RNA, which was consistent with other studies of COVID-19 [16, 17].

### Statistical analysis

Continuous variables were summarized as either means and standard deviations. Categorical variables were described as frequencies and percentages. The differences between the two groups were analyzed by the Fisher’s exact test or the Wilcoxon signed-rank test for categorical variables and the Mann-Whitney U test for continuous variables. We performed pairwise comparisons with a Bonferroni correction for multiple comparisons. The statistical adjusted significance was accepted at the adjusted *p* value < 0.0008.

The survival time was estimated by Kaplan-Meier method and Log Rank Test. Univariable Cox proportional hazard regression was performed to estimate associations between age, comorbidities and provenance from LSRCHs and clinical outcomes of mortality. Hazard ratios (HR), odds ratio (OR) and 95% confidence intervals (CI) were reported. A *p* < 0.05 was considered statistically significant. Age, comorbidities and provenance from residential care homes were used as predictors in a multivariable logistic regression model, with a binary outcome (mortality). Two model were created (Model 1: all of them; Model 2: only age). Calibration of agreement between the predicted and observed probabilities was assessed using the Hosmer-Lemeshow goodness-of-fit test, indicating a poor fit for *p* < 0.05.

All analyses were performed using the GraphPad Prism software (version 8.4.1) (GraphPad Software, San Diego, CA).

### Ethical approval

A written informed consent was obtained by participant to the study. The study was approved by our Institutional Ethical Committee (Sapienza University of Rome, Italy) (Prot.# 52SA_2020, RIF. CE 5773_2020), on the basis that it complied with the declaration of Helsinki and that the protocol followed existing good clinical practice guidelines.

## Results

### Clinical characteristics

A total of 81 consecutive patients, referred to our hospital for COVID-19 and identified as laboratory-confirmed SARS-CoV-2 infection, have been included in the study. They were 37 men and 44 women. Their median age is 79.7 years (ranging from 65 to 94 years). Demographic and clinical characteristics are listed in Table 1. On admission, most patients had shortness of breath (61%). There were differences in cough and fever between two groups. Both the respiratory scores (P/F and PSI) were higher in Very Elderly patients.

**Table 1:** Demographic and Clinical Characteristics

	Total (*n* = 81)	Age 65–79 (*n* = 41)	Age ≥ 80 (*n* = 40)	*p* value
Age (years)	79.69 ± 8.01	73.05 ± 4.71	86.5 ± 3.89	NS
Male (n, %)	37 (46%)	22 (53%)	15 (37%)	NS
**Symptoms**				
Cough	29 (35%)	20 (49%)	9 (22%)	0.01
Dyspnea	50 (61%)	24 (58%)	26 (65%)	NS
Fever	37 (45%)	25 (61%)	12 (30%)	0.005
**Respiratory Score**				
P/F	280.37 ± 113.64	316.58 ± 105.38	241.66 ± 110.95	0.009
PSI	121.59 ± 32.51	109.2 ± 27.62	134.3 ± 32.52	0.0003
**Medical history (n, %)**				
Neurological disease	42 (52%)	17 (40%)	25 (59%)	0.005
Cardiovascular disease	56 (69%)	23 (56%)	33 (82%)	0.01
Diabetes	22 (27%)	10 (24%)	12 (30%)	NS
Respiratory disease	28 (34%)	15 (36%)	13 (33%)	NS
Cancer disease	20 (25%)	12 (29%)	8 (20%)	NS
Chronic Kidney disease	16 (19%)	7 (17%)	9 (22%)	NS

Was used Bonferroni correction for multiple comparisons. The statistical adjusted significance was accepted at the adjusted *p* value < 0.0008

**Abbreviations:** *P/F* partial pressure of oxygen/inhalation volumetric fraction of molecular oxygen, *PSI* pneumonia severity index

Among comorbidities, the more representative were neurological and cardiovascular diseases. The incidence of both were more in Very Elderly patients.

### Laboratory results

All data obtained from Laboratory results are resumed in Table 2.

**Table 2:** Laboratory Results

	Total (*n =* 81)	Age 65–79 (*n =* 41)	Age ≥ 80 (*n =* 40)	*p* value
**Hemocromocytometric parameters**				
Haemoglobin (g/dl)	12.23 ± 1.81	12.57 ± 1.98	11.89 ± 1.56	NS
Platelet (10^3/uL)	211.07 ± 101.7	204.63 ± 99.4	217.33 ± 104.91	NS
WBC (10^3/uL)	8.37 ± 4.13	8.08 ± 4.82	8.66 ± 3.34	NS
Neutrophil %	74.1 ± 13.61	70.86 ± 14.97	77.33 ± 11.38	0.03
Lymphocytes %	18.07 ± 11.85	20.9 ± 13.41	15.23 ± 9.39	0.03
Monocytes %	6.72 ± 3.35	7.04 ± 3.05	6.39 ± 3.61	NS
Eosinophil %	0.85 ± 1.08	0.82 ± 1.04	0.88 ± 1.14	NS
Basophil %	0.27 ± 0.34	0.31 ± 0.41	0.23 ± 0.25	NS
Neutrophil (M/mm^3)	6.34 ± 3.36	5.86 ± 3.54	6.82 ± 3.14	NS
Lymphocytes (M/mm^3)	1.44 ± 1.71	1.67 ± 2.28	1.21 ± 0.76	NS
Monocytes (M/mm^3)	0.55 ± 0.37	0.55 ± 0.38	0.54 ± 0.35	NS
Eosinophil (M/mm^3)	0.41 ± 3.01	0.76 ± 4.26	0.07 ± 0.1	NS
Basophil (M/mm^3)	0.03 ± 0.08	0.03 ± 0.07	0.04 ± 0.09	NS
**Hormone Levels**				
FT4 (ng/dl)	1.1 ± 0.23	1.2 ± 0.27	1.03 ± 0.18	0.04
FT3 (pg/ml)	1.78 ± 0.55	1.81 ± 0.55	1.76 ± 0.57	NS
TSH (uU/ml)	1.01 ± 0.85	0.87 ± 0.81	1.11 ± 0.87	NS
Vitamin D 25-OH (ng/ml)	10.66 ± 9.57	8.4 ± 9.11	11.92 ± 9.84	NS
Ferritin (ng/ml)	843.4 ± 1110.26	692.15 ± 932.01	1006.76 ± 1274.7	NS
**Serum Markers of inflammation**				
Procalcitonin (ng/ml)	1.21 ± 3.92	0.53 ± 1.01	1.76 ± 5.15	NS
NLR	6.71 ± 5,44	5.35 ± 3.79	8.06 ± 6.47	0.02
PLR	206.76 ± 123.88	188.66 ± 101.18	223.89 ± 141.35	NS
C-Reactive Protein (mg/dl)	7.54 ± 7.28	5.53 ± 6.23	9.55 ± 7.76	0.02
**Blood Biochemistry**				
Creatinine (mg/dl)	1.23 ± 0.98	1.11 ± 0.63	1.35 ± 1.23	NS
Urea (mg/dl)	29.96 ± 25.22	26.33 ± 22.99	33.6 ± 27.07	NS
AST (U/L)	32.7 ± 36.23	31.35 ± 17.38	34.08 ± 48.79	NS
ALT (U/L)	25.09 ± 31.55	27.5 ± 21.21	22.62 ± 39.62	NS
LDH (U/L)	312.89 ± 134.51	294.41 ± 126.18	333 ± 142.16	NS
Alkaline phoshatase (U/L)	95.33 ± 50.86	92.48 ± 54.72	98.17 ± 47.74	NS
Troponin (pg/ml)	48.26 ± 81.84	36.48 ± 53.46	60.05 ± 102.46	NS
D-dimer (ng/ml)	974.93 ± 1270.89	861.18 ± 1270.87	1091.76 ± 1277.66	NS
**Serum Immunological Markers**				
T cells (%)	74.06 ± 11.43	76.31 ± 7.91	72.06 ± 13.77	NS
T cells (cells/uL)	735.76 ± 459.27	714.13 ± 291.77	755.00 ± 577.50	NS
B cells (%)	10.79 ± 6.05	10.88 ± 6.73	10.72 ± 5.58	NS
B cells (cells/uL)	111.71 ± 99.54	105.69 ± 95.79	117.06 ± 105.23	NS
T cells CD4+ (%)	48.26 ± 13.29	49.75 ± 12.01	46.94 ± 14.54	NS
T cells CD4+ (cells/uL)	505.35 ± 404.79	504.75 ± 334.32	505.84 ± 468.45	NS
T cells CD8+ (%)	23.82 ± 13.06	24.50 ± 10.97	23.22 ± 14.97	NS
T cells CD8+ (cells/uL)	209.85 ± 134.00	218.94 ± 118.10	201.78 ± 149.68	NS
NK cells (%)	14.25 ± 10.79	12.44 ± 7.72	15.83 ± 12.95	NS
NK cells (cells/uL)	129.06 ± 98.44	111.19 ± 74.55	144.94 ± 115.49	NS
CD4+/CD8+	2.81 ± 1.67	2.60 ± 1.36	3.01 ± 1.92	NS

Was used Bonferroni correction for multiple comparisons. The statistical adjusted significance was accepted at the adjusted *p* value < 0.0008

**Abbreviations:** *WBC* white blood cells, *M* thousand, *NLR* neutrophil to lymphocyte ratio, *PLR* platelet to lymphocyte ratio, *NK* natural killer

All of our COVID-19 patients, showed normal values of red blood cells (RBC), white blood cell (WBC) and platelets (PLT), measured on admission. Among the different serum markers of systemic inflammation and sepsis measured, we observed a higher value of the NLR and hs-CRP in Very Elderly compared to Elderly.

### Radiological findings

Results of the radiological evaluation of our elderly patients are reported in Table 3. On admission, the abnormalities in chest CT images detected in COVID-19 patients consisted in acute lung inflammatory lesions, involving one or multiple lobes. The proportion of bilateral and multiple lobes involvement is high in both groups and no difference was found. Most of the patients presented on CT scan ground glass opacity (82%), whereas pleural effusion has been found in 38.3% of them and difference between the two groups was significant.

**Table 3:** CT findings

	Total (*n =* 81)	Age 65–79 (*n =* 41)	Age ≥ 80 (*n =* 40)	*p* value
Monolateral (n, %)	13 (16.04%)	6 (14.6%)	7 (17.5%)	NS
Bilateral (n, %)	67 (82.71%)	34 (82.9%)	33 (82.5%)	NS
Single Lobe lesion (n, %)	13 (16.04%)	6 (14.6%)	7 (17.5%)	NS
Multiple Lobe lesion (n, %)	67 (82.71%)	34 (82.9%)	33 (82.5%)	NS
GGO (n, %)	66 (81.48%)	34 (82.9%)	32 (80%)	NS
Consolidations (n, %)	53 (65.4%)	25 (60.9%)	28 (70%)	NS
Pleural effusion (n, %)	31 (38.3%)	11 (26.8%)	20 (50%)	0.03

Was used Bonferroni correction for multiple comparisons. The statistical adjusted significance was accepted at the adjusted *p* value < 0.0008

**Abbreviation:** *GGO* ground glass opacity

### Outcomes

The duration of hospitalizations (LOS), measured in days, was longer for Very Elderly compared with Elderly but the difference is not statistically significant.

The median duration of viral shedding in the current study was 22 days. Based on the median as the cut-off point, the prolonged period of viral shedding was defined as a duration of more than 21 days. Among Very Elderly, 31 patients were still positive after 21 days respect 23 patients of Elderly patients (*p* < 0.05).

**Fig. 1:**
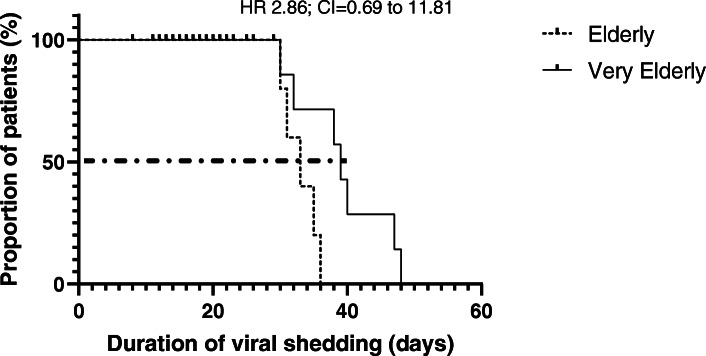
Kaplan-Meier curves representing the proportion of patients with detectable Viral RNA after 30 days from the onset of symptoms. (HR 2.86; 95% CI 0.69–11.81; *p* value = 0.03)

The Kaplan–Meier method was used to estimate the proportion of patients into two groups with a positive detection of SARS-CoV-2 RNA after 30 days from the onset of the symptoms (Fig.1). Very elderly patients showed a longer viral shedding compared to elderly one (HR 2.86; 95% CI 0.69–11.81; *p* value = 0.03).

**Fig. 2:**
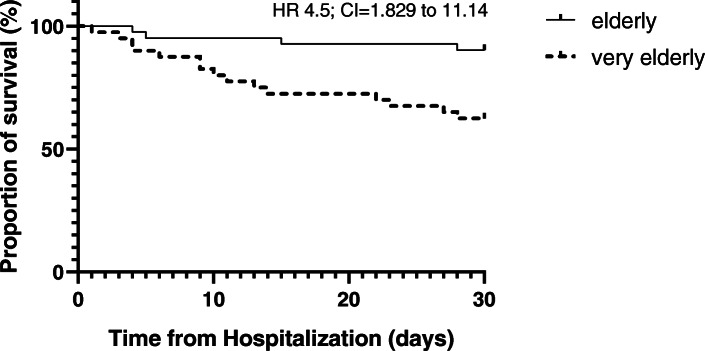
Kaplan-Meier survival curve for COVID 19 stratified by age. (HR 4.5; 95% CI 1.8–11.14; *p* value = 0.003)

By the end of May 19 patients (23%) died. Most of them belonged to the Very Elderly group. The mortality rate was significantly higher in Very Elderly (37.5%) than in Elderly (9.8%). Kaplan-Meier is shown in Fig. 2 (HR 4.5; 95% CI 1.8–11.14; *p* value = 0.003).

To explore the risk factors of death, univariate and multivariate logistics regression was conducted. Independently of age there are other factors that influence the outcome of COVID-19 patients. In particular, the presence and the number of comorbidities is a critical factor in predicting death in both elderly and very elderly patients. Another factor is the type of emergency admission to the hospital. A higher mortality rate was registered in patients admitted to the hospital from LSRCHs nearby Rome compared to those coming from their home and admitted directly via a general practitioner.

Table 4 reported the univariate logistic regression model suggesting that age (OR, 1.109; 95% CI: 1.031 to 1.206; *p* = 0.004), comorbidities (OR, 1.69; 95% CI: 1.124 to 2.652; *p* = 0.01) and provenience from LSRCHs (OR, 3.29; 95% CI: 1.057 to 12.58; *p* = 0.03) were a risk factors for death.

**Table 4:** Univariate Analysis

Variables	Univariate		
	OR	95%CI	*p* value
Age	1.109	1.031 to 1.206	0.004
Comorbidities	1.69	1.124 to 2.652	0.01
LSRCHs	3.29	1.057 to 12.58	0.03

**Abbreviations:** *OR* odds ratio, *CI* confidence interval

Table 5 showed the multivariate logistic regression analysis. It was found that the model with age, comorbidities and data regarding provenience from LSRCHs was preferred compared to the model that considered only age in predicting mortality (Hosmer and Lemeshow goodness-of-fit test, *p* = 0.79).

**Table 5:** Multivariate Analysis

Variables	Multivariate Model 1		Multivariate Model 2	
	OR	95%CI	OR	95%CI
Age	1.099	1.013 to 1.209	1.109	1.031 to 1.206
Comorbidities	1.732	1.084 to 2.864		
LSRCHs	2.168	0.599 to 9.088		

Hosmer and Lemeshow goodness-of-fit test, *p* = 0.79

**Abbreviations:** *OR* odds ratio, *CI* confidence interval

## Discussion

Elderly are generally defined as having a chronological age of 65 years or older [18]. In Japan, where many elderly people live, this definition was reviewed [19]. Thanks to the advances in medical and health science the lifespan has recently increased in Japan as well as in Italy. The simple chronological age appears to be no longer appropriate to the actual situation, in both Countries, where life expectancy is of 80 years and where there is an increased number of bright and energetic elderly people. Therefore, in Japan, the term of “late elderly” was introduced to indicate a new class of people, older than 75 years of age [18]. Other factors besides the chronological age appear to play a major role in affecting the health status and the expectance of life and a careful assessment of the “elderly frailty” is required to determine the biological, functional, cognitive and clinical aspects of the elderly subjects [20]. Previous reports indicate that elderly people are particularly susceptible to Community-Acquired Pneumonia [21–23]. When a community-acquired pneumonia is diagnosed in very elderly people, there is a significant increase in morbidity and mortality [24]. It has been observed that these patients often develop hospital-acquired complications and mortality occurs more frequently compared to younger people and to elderly ones. Moreover, the occurrence of pneumonia in elderly people is often the terminal event that complicates a long-term illness, such as dementia, cardiovascular disease, cancer, or prolonged immobilization syndrome [25]. However, it is not always easy to dissect the relative contribution of other factors, including disability, frailty, comorbidities and the health status of these patients, prior to the development of the disease. COVID-19 is a severe disease, caused by the SARS-CoV-2 virus, mostly affecting the lung where an interstitial viral pneumonia is frequently observed, with typical patchy bilateral ground glass opacities and peripheral consolidations. Elderly people appear to be more susceptible, especially to the more severe forms of the disease [26]. However, little information is available so far regarding the course of COVID 19 in very elderly people. It is well established that COVID-19 occurs more frequently among elderly people, with higher susceptibility to mortality and ICU admission [27], but a limited number of studies and of patients has focused the attention on a population over eighty years of age [3].

Our very elderly patients, showed a more severe disease, with higher level of serum marker of inflammation (hs-CRP and NLR) and higher severity respiratory indexes (PSI and P/F). This could be due to the increased inflammatory activity, associated with aging, reflected by increased circulating levels of TNF-alpha, IL-6, cytokine antagonists and acute phase proteins in vivo [28].

Experimental observations in mice, indicate that the SARS-CoV viral replication in aged mice is associated with clinical illness and pneumonia, demonstrating an age-related susceptibility to SARS disease in animals that parallels the human experience [29]. Moreover, they demonstrated that replication of SARS CoV is enhanced in aged mice compared to younger and enhanced viral replication is accompanied by evidence of clinical illness, alveolar damage, and interstitial pneumonitis [29].

Besides to be stroked by a more severe disease, median duration of viral shedding in our very elderly patients is higher compared to that recently reported in younger and symptomatic (14 days) or asymptomatic patients (19 days) [30] and there is a statistically significant difference between the two groups. This also affects the length of the stay (LOS) in the hospital which result higher in Very Elderly. We don’t have data regarding the immune response in terms of cytokine production and production of specific immunoglobulins in our very elderly patients. However, the increased duration of viral shedding suggests that they may have a weaker immune response to SARS-CoV-2 infection, compared to younger patients. Increasing age has been defined as a predictive factor for mortality in pneumonia patients in many studies, especially among patients aged 65 years or older [31, 32]. Several studies, suggested that age ≥ 85 years was an independent predictive factor for mortality in patients affected with community acquired pneumonia [33, 34]. Calle et al. reported that age ≥ 90 years was markedly associated with mortality [35]. Ageing is associated with a progressively weakened immune system and decreased lung performance. For patients of extreme age (≥ 85 years in our study), these changes alone are probably drastic, which independently increases the risk of death due to pneumonia [34]. In the study by Zunyou Wu et al., the overall case-fatality rate of those aged 70 to 79 years was 8.0% compared to aged 80 years and older where it was 14.8% [36]. However, in this study only 3% of the total number of cases were 80 years of age or older. In another study by Niu et al., the mortality rate in patients over 80 was equal to 18% [3]. Also Yan et al. reported that older patients (> 65 years) with comorbidities and ARDS are at increased risk of death, although even in this study patients over 80 years of age were a small number [37].

In our study the number of patients with more than 80 years of age is similar to that with 65–79. The mortality rate in Very Elderly was 37.5% and this percentage was significantly higher compared to that observed in Elderly. Our findings suggest that, similarly with other severe acute respiratory outbreaks, age is a fundamental risk factor for mortality. These results also emphasize the importance of the very advanced age (i.e. ≥ 85 years).

When we try to identify which factors other than age may influence the course and outcome of the disease in these patients, two conditions appear to play a major role. The first is represented by the pre-existing health conditions or comorbidities. Patients with pre-existing pathological diseases, and in particular those affected by multiple comorbidities die more frequently than those with no or few comorbidities. In other words, COVID-19, as other community-acquired pneumonias, acts as terminal event that complicates long-term illnesses. This is in agreement with previous studies demonstrating that the presence of any comorbidity is associated with increased risk of poorer clinical outcomes [38].

The second one consisted in the observation that patients admitted to the hospital coming from previous LSRCHs, were in worst clinical conditions and died more frequently compared to those admitted coming from their homes. There are many possible explanations to that. Patients in LSRCHs may suffer of more comorbidities and fragilities, and as previously observed they are more prone to accumulate complications, thus resulting in unfavorable outcome. Another explanation is that, staying in a close environment, makes these patients more susceptible to infections. In this regard, the results of the Italian National Survey indicated that one of the major difficulties was to obtain an adequate isolation of the positive patients. Finally, the disease could be more severe because of a delay in its recognition, that, according to this survey, was due to insufficient availability of the nasal/oropharyngeal tests. Considering all these factors and based on our observations, it is necessary to obtain a better evaluation of the frailties of elderly and very elderly people that represent a population more prone to the condition of risk and vulnerability, characterized by an unstable equilibrium, if facing negative events.

Because of the rapid evolving outbreak globally, ongoing studies with the inclusion of more patients would be needed to increase the statistical power of our results.

## Conclusions

In conclusion, the heightened susceptibility of the elderly to COVID 19, led us to demonstrate that there were considerable differences between Elderly and Very Elderly in terms of inflammatory activity, severity of disease and adverse clinical outcomes. Although age is one of the major risk factors for mortality, a thorough assessment of comorbidities and information about the provenience from residential care homes may help establishing risk stratification of patients with COVID-19 upon hospital admission and may furnish valuable information for planning adequate programs of intervention at the sanitary-assistential levels.

## Data Availability

The data that support the plots within this paper and other finding of this study are available from the corresponding author upon reasonable request.

## Abbreviation

LSRCHs: Long-stay residential care homes
